# Cardiovascular–Kidney–Metabolic Syndrome: A New Paradigm in Clinical Medicine or Going Back to Basics?

**DOI:** 10.3390/jcm14082833

**Published:** 2025-04-19

**Authors:** Victoria Mutruc, Cristina Bologa, Victorița Șorodoc, Alexandr Ceasovschih, Bianca Codrina Morărașu, Laurențiu Șorodoc, Oana Elena Catar, Cătălina Lionte

**Affiliations:** 1 Faculty of Medicine, Grigore T. Popa University of Medicine and Pharmacy, 700115 Iasi, Romania; cristina.bologa@umfiasi.ro (C.B.); victorita.sorodoc@umfiasi.ro (V.Ș.); alexandr.ceasovschih@umfiasi.ro (A.C.); morarasu.bianca.codrina@gmail.com (B.C.M.); laurentiu.sorodoc@umfiasi.ro (L.Ș.); 2 Second Internal Medicine Department, Sf. Spiridon Clinical Emergency Hospital, 700111 Iasi, Romania; 3 Department of Neurology, Centre Hospitalier Universitaire d’Angers, 49 933 Angers, Cedex 9, France; oanaelena.catar@chu-angers.fr

**Keywords:** hypertension, chronic kidney disease, diabetes, metabolic syndrome, obesity, heart failure, cardiovascular–kidney–metabolic syndrome

## Abstract

Cardiovascular, renal, and metabolic diseases are pathophysiologically interdependent, posing a significant global health challenge and being associated with a substantial increase in morbidity and mortality. In 2023, the American Heart Association (AHA) defined this complex network of interconnected health conditions as the cardiovascular–kidney–metabolic (CKM) syndrome. This syndrome is based on common pathophysiological mechanisms, including chronic inflammation, oxidative stress, hyperglycemia and insulin resistance, activation of the renin–angiotensin–aldosterone system (RAAS), and neurohormonal dysfunction, which trigger a vicious cycle where the impairment of one organ contributes to the progressive deterioration of the others. An integrated approach to these conditions, rather than treating them as separate entities, supports a holistic management strategy that helps to reduce the burden on public health and improve patients’ quality of life. Existing management focuses on lifestyle modification, glycemic and lipid control, and the use of nephroprotective and cardioprotective therapies. This narrative review aims to synthesize and contextualize existing information on the complex interactions between these systems and on diagnostic approaches, as well as to provide an overview of the available therapeutic options.

## 1. Introduction

We are witnessing an acceleration in the aging of the population, a phenomenon that requires sustained efforts to improve disease prevention and treatment strategies. Cardiovascular diseases (CVDs) remain the leading cause of death worldwide. Although mortality has declined among individuals over 65 years old, rates have remained stable among the younger population. This alarming trend highlights the need for early disease prevention measures focused on education, adopting a healthy lifestyle, and reducing risk factors [1]. Obesity, type 2 diabetes (T2D), CVDs, and chronic kidney disease (CKD) represent major global health challenges. According to the 2019 Global Burden of Disease Study, the prevalence of CKD and/or CVDs cases has doubled, increasing from 271 million in 1990 to 523 million in 2019 [2]. These conditions affected more than 26% of adults in the U.S. between 2015 and 2020 [3]. These diseases were once treated as separate entities; however, they have gradually been recognized as interconnected disorders sharing the same pathophysiological mechanisms that lead to their parallel progression. Cardiorenal syndrome represents the bidirectional relationship between CVDs and kidney dysfunction, which can lead to organ failure [4]. Cardiometabolic syndrome (CMS), in which excess adiposity is associated with the development of metabolic risk factors and metabolic syndrome (MetS) components, is linked to an increased risk of CVD and T2D [5]. The cardiac manifestation of the CMS is heart failure (HF), especially with a preserved ejection fraction (HFpEF) [6]. The incidence of HFpEF is expected to increase to an extent that will affect 1 in 10 individuals [7]. Additionally, studies have reported a connection between metabolic syndrome and CKD [8]. The synergistic relationship between these syndromes has created a cycle in which dysfunction in one system overlaps with and contributes to the impaired function of others. The entanglement of these systemic disorders led to the definition of cardiovascular–kidney–metabolic (CKM) syndrome (Supplementary Figure S1) by the American Heart Association (AHA) in 2023 [9]. CKM syndrome is described as a group of conditions associated with excessive/dysfunctional adipose tissue accumulation, and a CKM staging framework that reflects pathophysiology, risk spectrum, and opportunities for prevention and management is provided [9]. A poor CKM health system is a major determinant of morbidity and premature mortality. Interventions addressed to CKM syndrome are recognized as pivotal therapies for HF. Therefore, developing integrated strategies to improve CKM health throughout life is a key priority in the clinical and public health domains [10,11].

Several decades ago, the management of a patient with associated comorbidities in a unified manner was the task of internal medicine. The development of specialties derived from internal medicine, such as cardiology, nephrology, diabetology, and so on, was beneficial in studying several aspects of cardiovascular, kidney, or metabolic diseases in detail. However, a disadvantage ensued, in the sense that each specialist was more preoccupied with a certain disease or a narrow aspect of a disease particular to a certain organ or system and not with the entire condition of the patient. This separation in multiple planes of medical practice contradicts the unitary and indivisible character of the human body, where the organs and systems function not in an isolated manner, but in strict interdependence. At present, we see a plea to return to unified patient care, such as the development of nephrocardiologists (or cardionephrologists) to deal with cardiorenal syndromes [12]. In this context, should the integrated management of patients with CKM syndrome involve cardionephrologists and DM or obesity specialists, or should we create a new subspecialty of cardio-nephro-diabetology? We have to ask ourselves if internal medicine specialists could be a solution to this task, at least for the management of the patients in the in the initial stages of CKM syndrome. Although specialized care is needed for each component of CKM syndrome, the patients should be managed in a holistic manner, which can be done in internal medicine departments. This study proposes a holistic and integrated approach to managing overlapping diseases in the current cardiometabolic crisis by defining the syndrome, exploring its interconnected mechanisms, and outlining diagnostic and treatment strategies.

## 2. Epidemiological Evidence of CKM Disease Connections

Obesity, a disease officially recognized by the World Health Organization (WHO) in 1948 and by the European Commission in 2021, is characterized by excessive adiposity, with or without abnormal adipose tissue distribution or function, and multifactorial causes that remain incompletely understood [13,14]. Based on the present trends, overweight and obesity will affect nearly 3 billion adults (some 50% of the world’s adult population) by 2030 [15]. Obesity represents a major risk factor for developing CVDs, T2D, CKD, various forms of cancer, as well as multiple musculoskeletal and mental disorders [13,16].

Diabetes mellitus (DM) remains a substantial public health concern. According to the 2021 estimates of the International Diabetes Federation, over 529 million people aged 20–79 years had diabetes (11% prevalence), and this number is expected to rise to 783 million by 2045 [17,18]. Approximately 90% of individuals with diabetes have T2D, while 9–10% have type 1 diabetes (T1D) [19]. A meta-analysis of 102 prospective studies highlighted that individuals with T2D have a twofold increased risk of coronary artery disease (CAD), major stroke subtypes, and death attributed to other vascular causes [20]. This was further confirmed by the CAPTURE study, which estimated that approximately 25% of individuals with T2D also had CVDs [21].

Diabetic kidney disease (DKD) is a major complication of DM, affecting over one-third of the patients with T1D and nearly half of those with T2D [22]. It is the leading cause of end-stage kidney disease (ESKD) and represents the sixth leading cause of disability and the fourth leading cause of death worldwide [23].

HF is a heterogeneous clinical syndrome caused by cardiac injury and overload, leading to increased intracardiac pressure and insufficient cardiac output to meet the body’s needs [24]. There is a strong relationship between age and HF prevalence, with no significant sex differences: 2.1% in the adult population, 4.6% in those aged ≥50 years, and 10% in those aged ≥70 years [25]. Due to a decline in the estimated glomerular filtration rate (eGFR), nearly half of the HF patients also suffer from CKD [26].

These epidemiological data suggest a strong connection between obesity, T2D, HF, and CKD, with each condition contributing to the onset and progression of the others.

A recently published study highlights an alarming increase in the prevalence of CKD syndrome conditions during the COVID-19 pandemic. Compared to the pre-pandemic period (2017–2019), the percentage of people without CKD conditions decreased by more than 15% during 2020–2022. At the same time, the prevalence of T2D increased from 3.6% to 12%, a more pronounced increase than that reported in the U.S. (from 3.9% to 6.9%). In addition, combinations of CKD conditions became more common: “CKD plus T2D” and “CVD plus T2D” recorded significant increases, especially in men. Risk factors such as hypertension, dyslipidemia, and obesity increased by 14%, 20%, and 26%, respectively, while prediabetes increased by 170%. These data suggest that the pandemic has significantly amplified the pre-existing trends, highlighting the need for urgent measures to prevent and manage cardiometabolic risks [27].

## 3. Definition and Prevalence of Cardiovascular–Kidney–Metabolic Syndrome

CKM syndrome is defined as “a systemic disorder characterized by pathophysiological interactions between metabolic risk factors, the cardiovascular system, and CKD, leading to multiorgan dysfunction and a high rate of adverse cardiovascular outcomes” [9]. Individuals with metabolic risk factors, CKD, or both are prone to developing CVDs. CVDs may be associated with metabolic risk factors or CKD, and these entities may complicate the evolution of patients with CVDs. These are the patients who have CKM syndrome. The development of CKM syndrome and the occurrence of associated complications are strongly influenced by an unhealthy lifestyle and neglect of self-care, factors exacerbated by health policies, economic conditions, and unfavorable environmental factors [9]. A cross-sectional cohort study conducted on a sample of 11,607 participants in the National Health and Nutrition Examination Survey from 1999 to 2020 found that 25% of the study participants had at least one CKM-related condition, while CKM multimorbidity was observed in 8% of the participants. A higher comorbidity burden was associated with older age (incidence increasing in individuals aged over 65 years), male sex, racial/ethnic background, and socioeconomic characteristics [3]. Sex influences the prevalence and prognosis of CKM syndrome. A study conducted between 1988 and 2018 suggests that the overall prevalence of CKM syndrome has steadily increased in both men and women. However, the increase in the prevalence of CKM syndrome in stage 3 was more pronounced in men (from 18.9% to 22.4%) compared to women (from 13.9% to 15.2%). This trend is explained by the fact that men accumulate more visceral and hepatic fat than women, factors that contribute to an increased risk of DM and CKD [28].

## 4. Risk Factors for Cardiovascular–Kidney–Metabolic Syndrome

The progression of CKM syndrome is influenced by overweight or obesity, impaired glucose tolerance, hypertension, CKD, T2D, and hypertriglyceridemia. As the disease advances, the relative and absolute risk of CVDs, CKD, and mortality increases significantly. Beyond the factors mentioned above, additional risk factors are chronic inflammatory diseases such as psoriasis, rheumatoid arthritis, lupus, or HIV/AIDS, a family history of DM or kidney disease, mental health and sleep disorders, as well as elevated levels of high-sensitivity C-reactive protein (≥2.0 mg/L) [9,29].

Moreover, sex-specific factors, such as early menopause, pregnancy complications, polycystic ovary syndrome, or erectile dysfunction may influence disease progression [29]. Furthermore, the prevalence of these risk factors varies among ethnic and racial groups [30], including metabolic risk factors depending on the body mass index (BMI) value [19]. For example, individuals of South Asian ancestry have a higher proportion of ectopic body fat at any given BMI value [31].

Another major trigger of these differences is the impact of social determinants of health (SDOH), which include economic, social, environmental, and psychosocial aspects. These factors play a crucial role in the progression, diagnosis, and outcomes of patients with DM, CKD, and CVDs [32,33].

## 5. Pathophysiological Mechanisms That Underline the Cardiovascular–Kidney–Metabolic Connection

**Figure 1:**
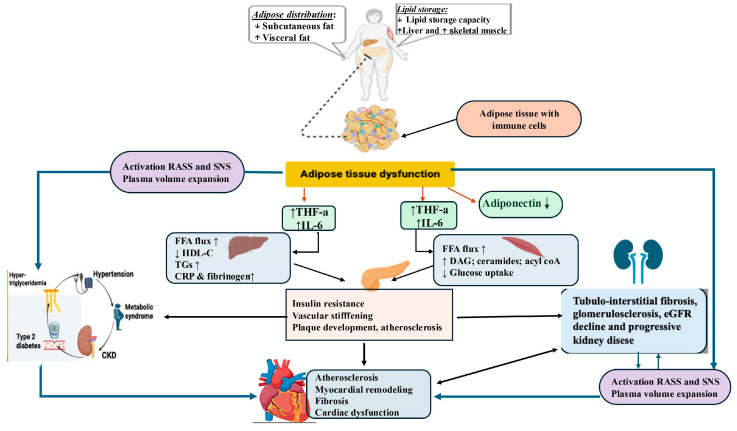
Selected pathophysiological mechanisms involved in CKM syndrome. FFA, free fatty acids; DAG, diacylglycerol; renin–angiotensin–aldosterone system; SNS, sympathetic nervous system; HDL-C, HDL cholesterol; TGs, triglycerides; CRP, C-reactive protein; CKD, chronic kidney disease; eGFR, glomerular filtration rate. Created with BioRender.com; accessed on 17 February 2025.

CKM syndrome is a progressive condition that begins early in life, influenced by factors such as early nutrition, environment, and social conditions, contributing to the excessive accumulation of adipose tissue in childhood [34]. The entities of the CKM syndrome are interconnected through common pathophysiological mechanisms, forming a vicious circle in which the dysfunction of one system exacerbates the abnormality progression in others. The pathophysiology of CKM syndrome is represented in Figure 1.

Obesity occurs when the energy intake consistently exceeds the caloric expenditure, leading to overmuch fat accumulation. Excess and dysfunction of adipose tissue, especially visceral fat, lead to increased infiltration of immune cells that secrete inflammatory and pro-oxidative molecules, such as cytokines (TNFα, IL-6) and free fatty acids (FFAs), which, when excessive, impair the function of insulin receptors and metabolic processes, leading to elevated glucose levels [35]. The released FFAs cause the liver to produce triglycerides and form very low density lipoproteins (VLDLs). This results in decreased levels of high-density lipoproteins (HDLs) and the appearance of small and dense low-density lipoprotein (LDL) particles, which are more atherogenic. Chronic inflammation, adipokine imbalance, and insulin resistance worsen this lipid profile, increasing the cardiovascular risk [36]. Obesity contributes to the development of hypertension through the activation of the sympathetic nervous system (SNS) and RAAS, a mechanism that induces endothelial dysfunction and reduces nitric oxide production. These pathophysiological changes lead to increased peripheral vascular resistance and heart rate, while the increased secretion of aldosterone promotes sodium retention, thus exacerbating hypertension. Furthermore, the chronic activation of the SNS and RAAS promotes the hypertrophy of smooth muscle cells in blood vessels, contributing to a further increase in blood pressure (BP) [37]. These mechanisms demonstrate the central role of obesity in the progression toward dyslipidemia, hypertension, and DM. Similarly, excess body weight is associated with obesity-related kidney disease (OKD), contributing to the onset or progression of CKD. The mechanisms involved include glomerulosclerosis, tubular inflammation, and renal fibrosis [38].

When DM and hypertension coexist, their impact on the kidneys becomes even more severe: chronic hyperglycemia and hypertension, through different mechanisms, lead to glomerular hyperfiltration in the early stages, which later progresses to the development of glomerulosclerosis, manifested by tissue hardening and scarring. Prolonged hyperglycemia stimulates the excessive production of reactive oxygen species, triggering oxidative stress, a key factor in organ damage in patients with DM. This activates several harmful metabolic pathways, including the polyol, hexosamine, and protein kinase C pathways and the formation of advanced glycation end products (AGEs). AGEs interact with receptors for advanced glycation end products on vascular and renal cells, contributing to tissue stiffening and the activation of pro-inflammatory and pro-fibrotic mechanisms [39,40].

The bidirectional heart–kidney relationship creates a vicious cycle sustained by hemodynamic changes and neurohormonal activations, which perpetuate a reciprocal damage [41]. Kidney and heart diseases are associated with mineralocorticoid receptor overactivation [42], with increased gene expression of NADPH oxidase, pro-inflammatory cytokines, and pro-fibrotic mediators [43]. These mechanisms lead, on the one hand, to glomerular damage, proteinuria, tubular injury, and reduced renal blood flow [44,45]. On the other hand, consequences on the cardiovascular system are vascular remodeling, endothelial dysfunction and increased vascular stiffness, as well as myocardial hypertrophy and ventricular remodeling/fibrosis, reduced coronary flow, and finally, myocardial injury (ischemia or infarction) [42,44,46]. HF leads to decreased cardiac output and renal hypoperfusion, which can cause chronic kidney damage and atrophy [26].

Cardiac dysfunction, particularly in HF patients with reduced ejection fraction (HFrEF), is correlated with excessive stimulation of neurohormonal systems (SNS and RAAS), which increases peripheral vascular resistance and affects the glomeruli, promoting proteinuria, fibrosis, and gradual nephron loss and accelerating CKD progression [26]. At the same time, sodium and water retention and persistent RAAS activation in CKD contribute to increased BP and post-load, leading to left ventricular overload. These disturbances can contribute to the development of left ventricular hypertrophy, myocardial fibrosis, wall stiffening, and the appearance of diastolic dysfunction [47]. The accumulation of uremic toxins (urea, indoxyl sulfate) in CKD patients affects endothelial function by reducing NO production and stimulates vasoconstriction, inflammation, atherosclerosis, and arrhythmogenesis, leading to pathological remodeling of the heart and worsening cardiac dysfunction [48].

The differential effects of sex hormones on cardiovascular, renal, and metabolic health are worth mentioning. Understanding and recognizing the impact of sex hormones on cardiac, renal, and metabolic health is essential for improving outcomes in patients with CKM syndrome. Estrogen demonstrates protective effects against renal injury and influences nephron structure and function. Moreover, estrogen enhances nitric oxide production and arterial dilation, thus preventing atherosclerosis and vascular injury. Testosterone’s influence suggests gender-specific mechanisms underlying the pathogenesis of CKD. Testosterone therapy tends to decrease inflammation, impacting vascular remodeling and the expression of adhesion molecules and growth factors. In the context of obesity and MetS, estrogen and testosterone influence fat distribution and metabolism, contributing to gender-specific differences in obesity prevalence [49].

## 6. Staging of Cardiovascular–Kidney–Metabolic Syndrome

**Figure 2:**
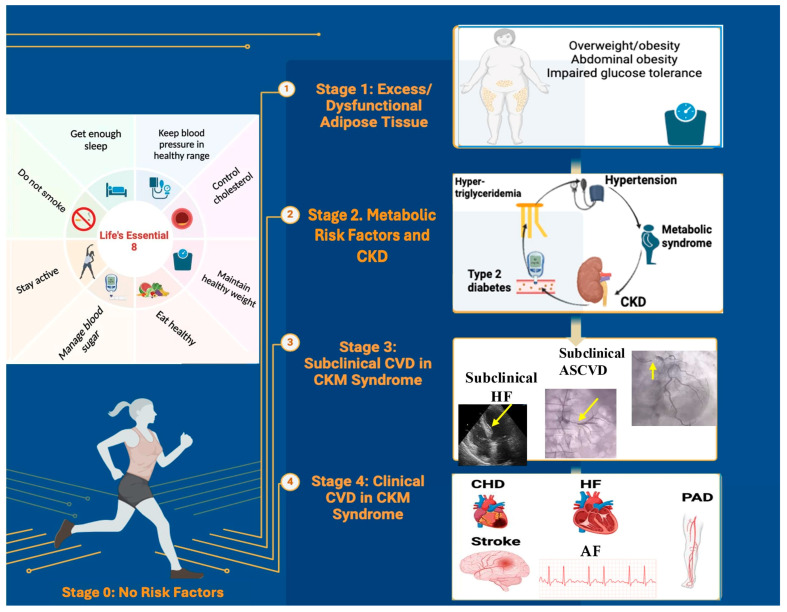
Stages of CKM syndrome, reflecting the pathophysiological progression and increased absolute risk of cardiovascular disease throughout the progression of this syndrome (adapted from [9], using images from personal collection). Stage 3 CKM Syndrome: yellow arrow shows, in the left image, a hypertrophied interventricular septum depicted in echocardiography, apical four-chamber view, as a marker of subclinical HF; in the middle image, yellow arrow shows left renal artery stenosis in renal angiography, as a marker of subclinical ASCVD; in the right image, yellow arrow shows a severe stenosis of the left main coronary artery in coronary angiography. Created with BioRender.com; accessed on 19 February 2025.

CKM syndrome is classified into four distinct stages, from stage 0 to stage 4 (Figure 2), which progressively increase the risk of developing CVDs. This staging system helps identify patients at different levels of CKM syndrome progression and severity, thus providing optimal opportunities to implement preventive measures aimed at halting or reversing disease progression.

### 6.1. CKM Syndrome Stage 0: No CKM Risk Factors

Stage 0 refers to individuals without cardiovascular risk factors, with normal BMI and waist circumference (WC), normal blood glucose, normal BP, a normal lipid profile, and no evidence of CKD or subclinical or clinical CVDs. The prevalence of stage 0 in women is 15.1%, and in men, 8.3% [28].

### 6.2. CKM Syndrome Stage 1: Excess and/or Dysfunctional Adiposity

The prevalence of CKM syndrome stage 1 in women is 27.1% and in men is 24.3% [28]. Stage 1 is divided into two subcategories.

Stage 1A: Excess adiposity manifested by overweight (BMI ≥ 25 kg/m^2^) or large normal WC (≥80/94 cm for women/men), or obesity (BMI ≥ 30 kg/m^2^) or abdominal obesity (WC ≥ 88/102 cm for women/men) according to the Centers for Disease Control and Prevention [50]. These intervals are used to assess the likelihood of developing obesity-related diseases [51,52]. However, there is evidence that the BMI predicts MetS differently in racial/ethnic groups. Asians present a higher risk of CVDs and T2D at a lower BMI compared with European populations. In this regard, the WHO has recommended lower anthropometric cutoff points for the Asian population (BMI ≥ 23–27.4 kg/m^2^ for overweight and ≥27.5 kg/m^2^ and above for obese, and WC ≥ 80/90 cm for women/men) [52].
Stage 1B: Dysfunctional adiposity manifested by the deposition of visceral and ectopic fat in organs such as the liver, heart, skeletal muscles, pancreas, and kidneys as a marker of higher cardiovascular risk independent of the BMI [53]. This is the consequence of abdominal obesity, which directly contributes to systemic inflammation and oxidative stress, impaired glucose tolerance, and prediabetes (Supplementary Table S1) [54,55]. A new evidence-based definition and diagnostic criteria for obesity were published in 2025. Preclinical obesity is defined as excess fat without evident organ dysfunction or functional limitations, but with a higher risk of progression towards clinical obesity and associated diseases. Clinical obesity is defined as a chronic systemic disease characterized by progressive organ damage and limitations in daily activities caused by excessive fat accumulation [13]. The visceral fat index has become a potentially useful parameter in evaluating the degree of visceral, hormonally active adiposity. The calculation of the visceral fat index is based on BMI, WC, triglycerides, and HDL cholesterol (HDL-C). It characterizes individuals with normal body weight but presents with a cluster of cardiovascular risk factors, including insulin resistance, impaired glucose tolerance, atherogenic lipid profiles, and hypertension, and is independently associated with an increased risk of HF and diastolic dysfunction [29].

### 6.3. CKM Syndrome Stage 2: Metabolic Risk Factors and CKD

In stage 2, the prevalence of CKM syndrome is almost equal (37.4% for women and 37.8% for men) [28]. Stage 2 is defined by the presence of metabolic risk factors (hypertension, hypertriglyceridemia, MetS, DM) and/or the presence of a low or moderate risk of CKD, according to the Kidney Disease Improving Global Outcomes (KDIGO) classification [9].

MetS is a multiplex risk factor for atherosclerotic cardiovascular disease (ASCVD) and T2D and is defined by the presence of three or more of the following: (1) WC ≥ 88 cm for women and ≥102 cm for men (≥80 cm for women and ≥90 cm for men if of Asian descent); (2) HDL cholesterol <40 mg/dL for men and <50 mg/dL for women; (3) triglycerides ≥150 mg/dL; (4) increased BP (SBP ≥ 130 mm Hg or DBP ≥ 80 mm Hg and/or the use of antihypertensive medications); (5) fasting glucose ≥100 mg/dL [5]. Abnormal glucose metabolism is classified into two distinct clinical categories: DM and prediabetes (Supplementary Table S1). DM should be suspected when symptoms such as polyuria, polydipsia, fatigue, blurred vision, weight loss, slow wound healing, and recurrent infections occur. However, the disease can be completely asymptomatic and remains undiagnosed in more than 40% of adults worldwide, with regional variations between 24% and 75% [19]. Diagnostic criteria were described by the WHO [56] and the American Diabetes Association (ADA) guidelines [57].

CKD is defined as an eGFR persistently <60 mL/min/1.73 m^2^ or a urinary albumin-to-creatinine ratio (UACR) ≥ 30 mg/g/ or ≥ 3 mg/mmol according to the latest KDIGO 2024 guidelines [58]. eGFR based on serum creatinine (eGFRcreatinine) and cystatin C (eGFRcystatin) are commonly used to assess kidney function. The difference between eGFRcystatin and eGFRcreatinine (eGFRdiff) is considered an independent indicator of kidney function. A study proved that an increase in eGFRdiff was associated with an improvement in CKM health, whereas a negative eGFRdiff tended to be associated with poorer CKM health. In addition, an increase in eGFRdiff was associated with a lower risk of all-cause mortality in CKM syndrome patients. These associations were independent of the UACR, and the eGFR best assessed kidney function [59].

### 6.4. CKM Syndrome Stage 3: Subclinical CVD

Stage 3 includes patients with subclinical ASCVD or subclinical HF in individuals with excess/dysfunctional adiposity and the presence of metabolic risk factors or CKD [9]. The subclinical CVD risk equivalents include a very high 10-year cardiovascular risk estimated with the risk scores (SCORE2, SCORE2-OP, or SCORE2-Diabetes) recommended by the European Society of Cardiology (ESC) and a high or very high CKD risk (according to the KDIGO classification).

Subclinical HF, as defined in the 2023 AHA/ACC guidelines, refers to patients in stage B of HF, representing an early stage of heart dysfunction characterized by structural and functional changes in the heart that are often asymptomatic but detectable through imaging tests or specific biomarkers [60]. The optimal method for identifying subclinical HF in the population is not yet fully clarified. However, elevated levels of cardiac biomarkers (B-type natriuretic peptide, BNP ≥ 35 pg/mL, or N-terminal pro-B-type natriuretic peptide, NT-proBNP ≥ 125 pg/mL) or persistently elevated high-sensitivity cardiac troponin levels (troponin T ≥ 14 ng/l for women and ≥22 ng/l for men, or troponin I ≥ 10 ng/l for women and ≥12 ng/l for men), as well as functional or structural heart abnormalities proved by echocardiography are now diagnostic criteria for subclinical HF [60]. The presence of both elements is associated with the risk of progression to clinical HF, as confirmed by the ARIC study [61]. Starting with stage 3, the prevalence of CKM in women begins to decrease compared to men (14.3% vs. 22.4%) [28].

### 6.5. CKM Syndrome Stage 4: Clinical CVD

Stage 4 CKM syndrome is defined by the presence of clinically manifest CVDs, including coronary artery disease (CAD), HF, stroke, peripheral arterial disease, and atrial fibrillation, in individuals with excess/dysfunctional adiposity and other risk factors such as CKD [9]. Stage 4 is further divided into stage 4a, referring to individuals without ESKD, and stage 4b, which includes individuals with ESKD. Based on the value of left ventricular ejection fraction (LVEF), HF has three phenotypes: HFpEF (LVEF ≥ 50%), HF with mildly reduced ejection fraction (HFmrEF, LVEF 41–49%), and HFrEF (LVEF ≤ 40%) [62]. The prevalence of stage 4 in women is 6.0% and in men is 7.2%. Although men have a higher prevalence of advanced-stage CKM syndrome, women have a higher risk of cardiovascular mortality [28].

## 7. Screening for Cardiovascular–Kidney–Metabolic Syndrome

The prevalence of CKM syndrome shows the need for effective biomarkers for CKM early detection and management. The screening for CKM syndrome should start early in life (<21 years old), with an assessment of overweight or obesity, BP, SODH, fasting lipid panel, plasma glucose, and other metabolic biomarkers. In adults (≥21 years old), it is necessary to additionally assess MetS components, including screenings for liver fibrosis, coronary artery calcium (CAC), subclinical HF, and renal impairment [63]. Systematic evaluation and appropriate clinical staging of the CKM syndrome are essential for early interventions and the prevention of disease progression. In this regard, we can obtain a comprehensive picture of the patient’s metabolic state and overall health.

Obesity screening should include, in addition to the BMI, at least one additional anthropometric measure, such as WC or the direct measurement of body fat. This is particularly important for athletes, individuals with a high muscle mass, or those with a BMI near the obesity threshold. For very high BMI values (>40 kg/m^2^), obesity can be presumed without additional evaluations [13]. A valuable biomarker could be leptin, an amino acid synthesized by white adipose tissue, which regulates appetite. It is a vascular tone mediator and can repair the myocardium tissue to normal levels. High levels of serum leptin have been associated with the pathogenesis of CVDs and MetS factors. Leptin has direct and indirect effects on the kidney leading to an impaired renal function, with a decreased GFR and albuminuria [63].

Cardiovascular evaluation should begin with the regular measurement of BP, either ambulatory or at home. According to the ESC guidelines [64], for patients with CKM syndrome stages 0 and 1, BP should be <110/70 mmHg. Patients are considered hypertensive if their SBP is >140 mmHg and DBP is >90 mmHg [64]. A recent study showed that even younger patients who maintain good health, are asymptomatic, and have no known CVD, CKD, DM, or hypertension have evidence of subclinical CVDs in 9% of the cases analyzed. The two traditional cardiac biomarkers (hs-cTnT and NT-proBNP) were significantly associated with CVDs and all-cause mortality; thus, monitoring only traditional, large macro-level risk factors may be insufficient for risk stratification, and further advances in precision medicine may be needed to identify the CVD risk in younger populations [65].

Periodically determining FPG and A1c and, if necessary, performing the OGTT (Supplementary Table S1) and Homeostatic Model Assessment of Insulin Resistance (HOMA-IR) allow for the detection of glucose metabolism impairments. The monitoring of FPG or A1c varies depending on the patient’s stage: once every 3–5 years for those in stage 0, every 2–3 years for those in stage 1, and annually or once every 2 years for those in stage 2 [66]. A new index for evaluating insulin resistance is the triglyceride–glucose (TyG) index, based on the fasting glucose and triglyceride levels. As the TyG index increases, the likelihood of developing advanced CKM syndrome also increases [67]. The modified TyG indices, which involve TyG, BMI, WC, and waist-to-height ratio combined, were confirmed to have better predictive ability than the TyG index for CVDs. The TyG index and modified TyG indices combined with obesity indicators proved to have positive linear relationships with CVD incidences in people with CKM stages 0–3. Monitoring and managing the modified TyG indices may be important in the early detection and intervention in the context of CKM syndrome [68].

The lipid profile assessment (i.e., total cholesterol, LDL-C, HDL-C, triglycerides, and lipoproteins) enables the early detection and management of metabolic abnormalities, thus preventing the risk of ASCVD. The atherogenic index of plasma (AIP), calculated as the log-transformed ratio of TGs to HDL-C in molar concentrations, a biomarker for plasma atherosclerosis, has emerged as a superior predictor of the CVD risk compared with individual lipid markers. Numerous studies in recent years have validated the association between the AIP index and the risk of various conditions, including DM, prediabetes, hypertension, MetS, myocardial infarction, and non-alcoholic fatty liver disease (NAFLD) [69,70,71]. Notably, a recent study showed a correlation between AIP and an increased incidence of CVDs in individuals with CKM syndrome stages 1–3; therefore, AIP should be considered a straightforward indicator of CVD, and individuals with elevated AIP levels, particularly those with CKM syndrome stage 3, should prioritize CVD prevention [72]. The measurement of lipoprotein(a) [Lp(a)] is essential for assessing the CV risk, as epidemiological and genetic studies confirm a causal relationship between elevated levels of Lp(a) and major CV events. The European Atherosclerosis Society recommends measuring Lp(a) at least once in adulthood and that the results be interpreted in the context of the patient’s overall CV risk. Identifying elevated Lp(a) levels allows for early interventions, including lifestyle modifications and intensification of risk factor management, which help reduce the incidence of ASCVD [73].

Another biomarker that is useful for the early screening of CKM syndrome is serum uric acid. Hyperuricemia is strongly associated with atherosclerosis, CVD, CKD, obesity, T2D, and NAFLD [74]. Studies show that hyperuricemia may be an independent factor for mortality and morbidity due to CVD, acute coronary syndrome, stroke, and HF [75]. Additional possible biomarkers for CKM syndrome are bilirubin, soluble neprilysin, lipocalin-type prostaglandin D synthase, and endocan. Bilirubin, which has antioxidant effects, improved cardiorenal and metabolic dysfunction and was associated, in high levels, with improved left ventricular remodeling. In this regard, it might be useful in CKM syndrome staging [63].

Inflammatory indices have been correlated with different stages of CKM syndrome. The systemic immune–inflammation index (SII) can be calculated from platelet, neutrophil, and lymphocyte counts, providing a measure of immune response and systemic inflammation. The SII offers insights into the inflammation underlying chronic diseases affecting cardiovascular, renal, and metabolic systems and helps monitor treatment responses. Studies have shown that the SII is significantly associated with various components of CKM syndrome [76]. Advanced CKM stages and elevated systemic immune–inflammation index (SIRI) are each independently associated with increased risks of all-cause and CVD mortality. The SIRI is calculated using neutrophil, monocyte, and lymphocyte counts. Individuals with both advanced CKM stages (stage 3 or 4) and a SIRI > 0.81 exhibited the highest mortality risks, particularly pronounced in adults under 60 years of age. These results highlight the potential value of integrating inflammatory markers such as the SII and SIRI into CKM syndrome risk stratification models, which offers clinicians more precise tools for identifying high-risk individuals and optimizing interventions [77].

Additionally, renal function tests, including eGFR and UACR determination, should be performed annually in patients with T1D, starting 5 years after their diagnosis, and at least once a year in individuals with T2D, from the first visit, according to the ADA recommendations [78]. For individuals with established CKD, these tests should be conducted 1–4 times a year, depending on the stage of renal disease [79]. Additional studies of patients with CKM syndrome are needed to determine how broadly and frequently to screen patients for albuminuria, whether it is cost-effective to treat low-grade albuminuria (10–30 mg/g), and how to equitably offer newer antiproteinuric therapies across the spectrum of CKM diseases [80].

Given the presence of metabolic disorders as a determining factor in the development of CKM syndrome, it is crucial for specialists to check for hepatic steatosis associated with metabolic dysfunction (MASLD), previously known as NAFLD. The criteria for its diagnosis include evidence of hepatic steatosis, plus one of the following three criteria: overweight/obesity, presence of T2D, or signs of metabolic disturbance [81]. Annual monitoring is necessary for patients with obesity and T2D and involves using transaminase measurement and liver elastography to determine the degree of fibrosis. This helps identify the disease before it progresses to non-alcoholic steatohepatitis associated with metabolic dysfunction, fibrosis, cirrhosis, and hepatocellular carcinoma [82].

Screening for subclinical HF can be performed using echocardiographic evaluation and biomarker measurement. In addition to the traditional biomarkers used for HF diagnosis, new cardiac biomarkers are emerging. ST2, a member of the interleukin-1 receptor family, is considered a new biomarker for heart disease and plays a crucial role in HF, providing independent and complementary prognostic information alongside NT-proBNP. Levels of ST2 > 35 ng/mL are associated with adverse outcomes and increased mortality, regardless of LVEF. Serial measurements of ST2 add additional prognostic value, and its interaction with HF therapies suggests potential for guiding treatment. Further studies are needed to validate the extended clinical use of this biomarker [83]. Other biomarkers associated with an increased risk of heart failure include galectin-3, GDF-15, IL-6, and procalcitonin [84]. The new biomarkers offer a more precise and timely diagnosis of HF, improving prognosis and risk stratification.

**Figure 3:**
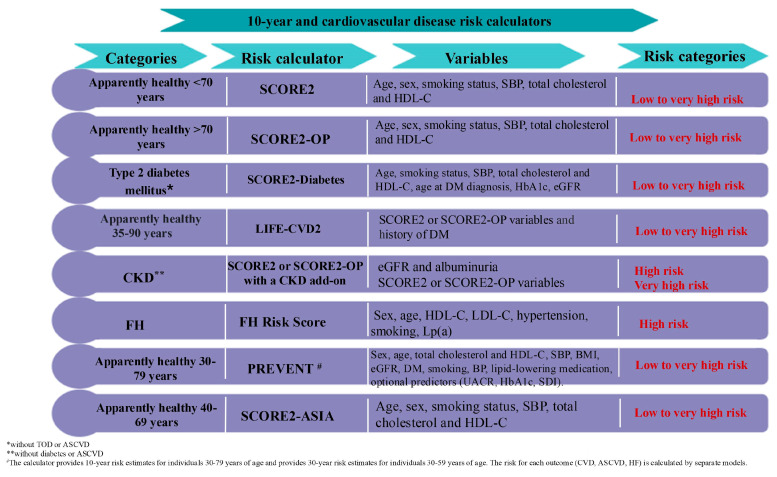
Selected risk calculators needed for screening CKM syndrome. ASCVD, atherosclerotic cardiovascular disease; eGFR, glomerular filtration rate; HbA1c, hemoglobin A1c; BP, blood pressure; SBP, systolic blood pressure; HDL-C, HDL cholesterol; OP, older persons; TOD, target organ damage; CKD, chronic kidney disease; FH, familial hypercholesterolemia; Lp(a), lipoprotein(a); BMI, body mass index; UACR, urinary albumin-to-creatinine ratio; SDI, social deprivation index.

The ESC recommends the use of prediction models (Figure 3) to estimate the 10-year risk of fatal and non-fatal cardiovascular events (such as myocardial infarction and stroke). SCORE2 is recommended for European patients without prior CVD or DM, aged 40–69 years [85]. SCORE2-OP is used in primary care settings for individuals aged over 70 years without a known CVD to estimate the 10-year risk for non-fatal or fatal CVDs in four geographical risk regions [86]. SCORE2-Diabetes is a recalibrated prediction model for estimating the 10-year risk of CVD in individuals under 70 years of age who have a history of DM in Europe [87].

SCORE2 and SCORE2-OP risk assessment models now have CKD add-ons based on data from the Chronic Kidney Disease Prognosis Consortium, which improve CVD risk prediction beyond SCORE2 and SCORE2-OP. This approach will help clinicians and patients with CKD refine patient risk prediction and further personalize preventive therapies for CVDs [88]. The SCORE2-ASIA model was recently developed for the accurate prediction of the 10-year risk of CVD in apparently healthy people in Asian countries, thereby enhancing the identification of individuals at higher risk of developing CVDs across the Asia Pacific Region [89].

For North American individuals aged 30–79 years without a known CVD, the AHA/ACC developed the Predicting Risk of Cardiovascular Disease Events (PREVENT) calculator [9], which allows for the prediction of the 10- and 30-year risk of CVD and CVD subtypes, including ASCVD, HF, CAD, and stroke. The PREVENT equation incorporates factors associated with CKM and the SDOH [90]. The new calculator includes CKM conditions alongside traditional risk factors such as smoking, BP, and cholesterol. For the first time, the HF risk is integrated into the total CVD risk assessment and can be calculated separately, considering its higher prevalence in individuals with DM and/or CKD [90]. Given that few young people have a high 10-year CVD risk, the new equation includes 30-year risk estimates starting from the age of 30, which are particularly useful for counseling and early prevention. A similar initiative for assessing the HF risk for European individuals would be beneficial. This evaluation helps identify individuals at high risk, allowing for early and personalized interventions. The 10-year risk of CVD ranges from low (<10%) to very high (30%) (Figure 3).

With the worldwide life expectancy continuing to rise, the LIFEtime-perspective CardioVascular Disease 2 (LIFE-CVD2) model for estimating lifetime risk and CVD-free life expectancy in individuals without previous CVD or DM in four European risk regions was recently developed. LIFE-CVD2 can be applied to individuals with a current age between 35 and 90 years and improves the stability of estimates in people of all ages with a very high life expectancy. Moreover, LIFE-CVD2 is well adapted to the contemporary clinical practice for both sexes [91].

Patients with established ASCVD have a 10-year absolute risk of 20% or more of experiencing recurrent vascular events such as cardiovascular death, ischemic stroke, or myocardial infarction. Residual risk of CVD is defined as the risk of ASCVD events that persist despite treatment or achieving targets for risk factors such as LDL cholesterol, BP, and glucose levels [69]. Risk stratification tools for secondary prevention to predict the long-term risk of recurrent vascular events include the Secondary Manifestations of Arterial Disease (SMART) score (Supplementary Figure S2) and the European Action Model for Secondary and Primary Prevention through Intervention to Reduce Events (EUROASPIRE) risk model [85].

When CVD risk scores are imprecise or if there is uncertainty regarding the benefits of pharmacological therapy for patients at moderate risk, additional tests for detecting subclinical atherosclerosis may be useful. The most studied imaging methods for screening subclinical atherosclerosis are coronary artery calcium (CAC) scoring, carotid intima-media thickness assessment, and evaluation of carotid artery plaque burden [92]. The CAC score is a highly specific marker of atherosclerosis that can be quantified using non-contrast computed tomography. It evaluates the risk of ASCVD based on the amount of calcium in the arterial walls, serving as a marker for atherosclerotic plaques. It can be useful for patients with low to moderate calculated 10-year CVD risk and can be repeated approximately every 5 years in those with very low or normal CAC scores [93].

Additionally, individuals with CKM syndrome should be encouraged to use validated applications, such as watches, smartphones, and tablets, which have shown promising results in improving physical health and weight management, particularly in terms of physical activity regulation, reducing sedentary behavior and promoting weight loss [94].

This multidimensional approach allows for early intervention and can slow down or even halt the progression to advanced CVD, CKD, or cirrhosis, ultimately improving long-term outcomes.

## 8. Management of Cardiovascular–Kidney–Metabolic Syndrome

*Stage 0*: The management of patients in stage 0 involves education and the implementation of a healthy lifestyle aimed at maintaining cardiovascular health. For this, the AHA [95] has proposed Life’s Essential 8 (Supplementary Figure S3). Maintaining these optimal components has been associated with greater survival without CVD, longer overall longevity, and better quality of life [95].

*Stage 1*: The management of CKM syndrome stage 1 focuses on reducing excessive or dysfunctional adiposity to prevent the emergence of metabolic risk factors (Supplementary Figure S4). The treatment strategies are based on the principle that the number of calories consumed should not be greater than the number of calories burned. Behavioral changes, the implementation of hygiene dietary measures, and adapted physical activity are used as first-line treatments for obesity. The Mediterranean diet, which is high in unsaturated fats, is associated with a weight loss of 5–10%, while physical activity has modest effects on weight reduction but is important for maintaining weight loss and reducing the CV risk [14], all of which are adapted to the patient’s medical conditions. If sufficient weight loss cannot be achieved, the addition of anti-obesity medications (AOMs) in conjunction with lifestyle modifications for overweight or obese patients, in the presence of at least one weight-related comorbidity, may be considered. AOMs have been shown to reduce the cardiometabolic risk [14]. Currently, there are six medications approved by the European Medicines Agency and the Food and Drug Administration for long-term obesity management: orlistat, an extended-release naltrexone/bupropion combination, the glucagon-like peptide 1 receptor agonists (GLP-1RAs) liraglutide and semaglutide, and the dual glucose-dependent insulinotropic polypeptide/glucagon-like peptide-1 receptor agonist (GIP/GLP-1RA) tirzepatide [14].

A weight loss of at least 5%, with increased benefits for losses of 10%, improves glycemic control, lipid levels, and BP in overweight and obese adults with T2D [19,96,97]. The American Association of Clinical Endocrinologists (AACE)/American College of Endocrinology (ACE) recommends targeting a 2.5% weight loss in the first month for patients with overweight or obesity [97], while the ACC/AHA/The Obesity Society (TOS) recommends a 5–10% weight loss within 6 months [98]. GLP-1RAs have revolutionized the treatment of obesity, offering significant weight reductions and a decrease in cardiovascular events in individuals with obesity and CVDs [99]. The first studies proving this benefit involved liraglutide, which demonstrated a mean weight loss of 8% vs. that of 2.6% achieved with placebo [14,100]. In diabetic patients, liraglutide, 3.0 mg, led to significantly greater weight loss (−6.0%) compared to placebo (−2.0%). Over 54% of the treated patients lost at least 5% of their initial weight, and 25.2% lost more than 10% [101]. More recently, studies with semaglutide (Table 1) showed, along with significant weight loss, improvements in cardiometabolic risk factors, such as WC, BP, and lipid profiles [102].

Tirzepatide, a dual GIP/GLP-1 RA, sets a new standard in obesity management, being extremely effective for weight loss (Table 1). In the long term, tirzepatide reduced the risk of T2D by 93% and maintained a weight loss of 17.8% even after stopping therapy [109].

Orlistat and bupropion/naltrexone should be cautiously used in patients with CVD due to their modest effects on weight and long-term cardiovascular risks [14].

If weight is not effectively managed through lifestyle interventions and medication, endoscopic intragastric interventions and bariatric surgery should be considered. Indications for endoscopic procedures are a BMI ≥ 30 kg/m^2^ up to <40 kg/m^2^ or a BMI > 27 kg/m^2^ in patients with one or more obesity-related comorbidities. These procedures reduce the gastric capacity, delay gastric emptying, and increase satiety [122]. Despite the emergence of endoscopic procedures (Supplementary Figure S4), bariatric surgery remains the most effective treatment for obesity in terms of weight reduction, improvement of comorbid conditions, enhancement of quality of life, and reduced patient mortality [122]. Bariatric surgery should be considered for patients with T2D and a BMI ≥ 35 kg/m^2^ or in patients with a BMI ≥ 40 kg/m^2^ [14].

**Figure 4:**
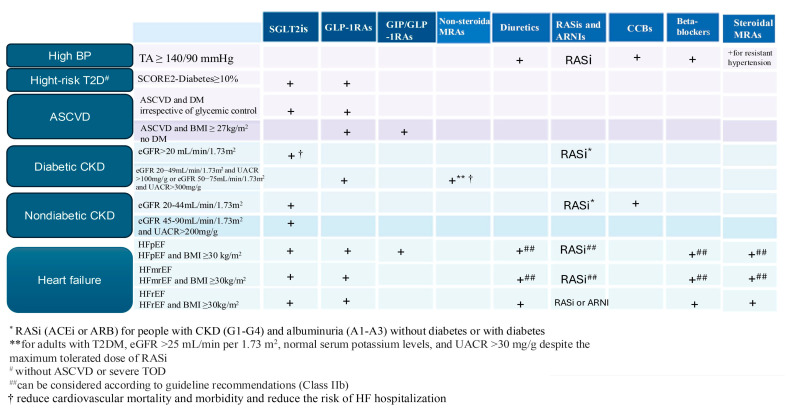
Algorithm for the use of SGLT2is, GLP-1RAs, and GIP/GLP-1RAs in managing CKM syndrome according to available guidelines, including other therapies that should be tailored to each patient. BP, blood pressure; ASCVD, atherosclerotic cardiovascular disease; BMI, body mass index; CKD, chronic kidney disease; DM, diabetes mellitus; eGFR, estimated glomerular filtration rate; GIP, glucose-dependent insulinotropic polypeptide; GLP-1RA, glucagon-like peptide-1 receptor agonist; HF, heart failure; HfmrEF, heart failure with mildly reduced ejection fraction; HfpEF, heart failure with preserved ejection fraction; HfrEF, heart failure with reduced ejection fraction; SCORE2, Systematic Coronary Risk Evaluation 2; SGLT2is, sodium–glucose cotransporter-2 inhibitor; TOD, target organ damage; T2DM, type 2 diabetes mellitus; UACR, urinary albumin-to-creatinine ratio; RASi, renin–angiotensin–system inhibitor; ACEi, angiotensin-converting enzyme inhibitor; ARB, angiotensin II receptor blocker; ARNi, angiotensin receptor/neprilysin inhibitor; steroidal MRAs, mineralocorticoid receptor antagonists (spironolactone and eplerenone); non-steroidal MRAs, mineralocorticoid receptor antagonist (finerenone). Created with BioRender.com; accessed on 22 February 2025.

*Stage 2*: Starting from this stage of CKM syndrome, management involves the appropriate use of cardioprotective and nephroprotective therapies based on the components of MetS, T2D, and CKD, with the aim of preventing ASCVD and its progression to subclinical or clinical CVD. Reducing the cardiovascular risk involves lifestyle modifications, followed by targeted pharmacological treatment for controlling BP, dyslipidemia, and diabetes and preventing the onset of CKD. A proposed algorithm for the management of patients with CKM syndrome stages 2 to 4 is presented in Figure 4.

The management of arterial hypertension should follow the recommendations set by current guidelines [64], starting with lifestyle modifications, adopting a balanced diet with a low sodium content, and using pharmacotherapy as necessary. The current guidelines recommend a target BP for those under treatment, with SBP between 120 and 129 mmHg and DBP between 70 and 79 mmHg [64]. A surprising effect of reduced BP has been observed as a result of treatment with sodium–glucose cotransporter-2 inhibitors (SGLT2is) [123]. SGLT2is lower BP through osmotic diuresis and natriuresis, reducing plasma volume and preload. Their long-term use may decrease sympathetic activity and improve arterial compliance. GLP-1RAs might also represent a hope for hypertensive patients. In the SURMOUNT-1 study, tirzepatide significantly reduced BP through indirect effects related to weight loss and improved glycemic control, and 58% of the participants on tirzepatide reached normal BP values [124].

In patients with T2D, antihyperglycemic therapy should be personalized according to each patient’s individual glycemic control objectives. The ADA and the European guidelines recommend an A1c target of <7.0% to reduce the risk of microvascular complications in most patients. A less stringent goal of A1c < 8.0% is recommended for older, frail adults with multiple comorbidities and shorter life expectancy [19,125]. Based on studies demonstrating the benefits of SGLT2is or GLP-1RAs [126,127], the 2023 ESC guidelines recommend a staged approach in managing patients with T2D [19]. For patients at high cardiovascular risk, with established ASCVD, and those with CKD and/or HF, a class I recommendation was made for these agents, regardless of other antihyperglycemic or anti-diabetic medications or A1c goals [19]. For patients with a high CV risk (SCORE2-Diabetes > 10%) but without ASCVD or target organ damage (TOD), metformin is recommended, with class IIb recommendations for the use of SGLT2is and GLP-1 RAs [19]. The treatment of patients with T2D with SGLT2is, including canagliflozin, dapagliflozin, empagliflozin, and sotagliflozin, may be prioritized for those with CKD due to their protective effects on renal function decline (Table 1), HF hospitalization, major cardiovascular events (MACEs), and cardiovascular death [60,117,118], while GLP-1 RAs are recommended for those with BMI ≥ 35 kg/m^2^, A1c ≥ 9%, or taking high insulin doses, as they offer additional cardiovascular benefits [106,128]. The combination of metformin with SGLT2is or GLP-1RAs is recommended for patients with A1c ≥ 7.5% to achieve glycemic targets [9,128]. Metformin is not a prerequisite for initiating therapy with SGLT2is or GLP-1 RAs, since these agents offer clear cardiovascular and renal benefits [19].

New agents, such as the triple-hormone-receptor agonist retatrutide, are studied. Retatrutide is an anti-diabetic agent with triple agonistic activity targeting glucagon, glucagon-like peptide-1, and gastric inhibitory polypeptide. Clinical trials investigated retatrutide’s role in the treatment of obesity and MASLD, as well as the effects of this therapy on cardiovascular outcomes, and renal functions [129,130,131].

Lipid parameters are crucial in preventing CV risk. In the primary prevention of ASCVD, individuals aged 40–75 years, with an LDL-C of 70–190 mg/dL and at intermediate risk (7.5–20%), should be started on moderate-intensity statin to reduce LDL-C by 30–49% (class I recommendation). In selected adults, if the risk estimate is uncertain, the CAC score helps the decision regarding statin (i.e., for individuals aged over 55 years, a CAC score of 1–99 favors statin therapy) [132]. In secondary prevention, continuous monitoring should occur until the LDL-C levels drop by at least 50% from the baseline or when the patient reaches their cholesterol target, recommended based on the CV risk profile. European guidelines for CVD prevention recommend an ultimate LDL-C goal of <55 mg/dL for apparently healthy persons < 70 years at very high risk and an LDL-C goal of <70 mg/dL in persons at high risk [133]. Given that most individuals with diabetes are at intermediate or high risk for ASCVD, statin therapy is also recommended [9,19]. Achieving these levels often requires a combination therapy (statins, ezetimibe, PCSK9 inhibitors, and bempedoic acid) [19,118]. Hypertriglyceridemia increases the risk of ASCVD and is often associated with MetS and insulin resistance [9]. After excluding secondary causes, lifestyle changes are the initial treatment. For residual hypertriglyceridemia in patients at intermediate or high ASCVD risk, statin therapy is recommended to reduce the ASCVD risk and moderately lower triglycerides (by 10–30%). For patients with triglycerides ≥ 500 mg/dL, who are at risk of pancreatitis, fibrate therapy, preferably fenofibrate, is recommended to be associated with statins. For patients with triglycerides between 135 and 499 mg/dL, with DM and additional risk factors, icosapent ethyl can be used to reduce the ASCVD risk [9].

DM and hypertension increase the risk of CKD. In turn, CKD amplifies the risks of ASCVD, HF, arrhythmias, hypoglycemia, and premature mortality. All patients with CKD should receive the maximum tolerated dose of a renin–angiotensin–aldosterone system inhibitor (RAASi), such as an angiotensin-converting enzyme inhibitor (ACEi) or an angiotensin II receptor blocker (ARB), and an SGLT2i, regardless of the presence of diabetes [58,80,134]. Studies have shown that SGLT2is are the cornerstone therapy for CKD (Table 1), regardless of DM presence, for reducing both renal and cardiovascular risk [108,109,110,111,112]. When starting treatment with RAASis or SGLT2is, a decline in eGFR is expected, manifested as mild to moderate increases in serum creatinine: ≤30% for RAASis, and 3–10% for SGLT2is. Neither RAASis nor SGLT2is should be discontinued in the absence of signs of extracellular volume depletion [134]. According to the 2024 KDIGO guidelines, SGLT2is have a class 1A recommendation for all patients with T2D, CKD, and eGFR ≥ 20 mL/min/1.73 m^2^. Additionally, SGLT2is are recommended for non-diabetic CKD patients with eGFR ≥ 20 mL/min/1.73 m^2^ and UACR > 200 mg/g (≥20 mg/mmol) and for those with HF, regardless of their albuminuria levels [58]. For patients with DKD, with eGFR > 25 mL/min/1.73 m^2^ and UACR > 30 mg/g, treated with the maximum tolerated doses of ACEis/ARBs, a non-steroidal MRA, finerenone, should be considered in addition to SGLT2i [119]. Finerenone is recommended for preventing HF hospitalization in patients with CKD and T2D, as per the updated ESC HF guidelines [24]. Two major studies (Table 1) have demonstrated the efficacy of this medication in reducing renal failure and DKD progression and in reducing cardiovascular mortality and morbidity in DKD patients [119].

*Stage 3*: The goal of management in stage 3 focuses on preventing disease progression through intensive interventions in individuals with subclinical HF or high-risk CKD, aiming to reduce disease progression to clinical HF and advanced CKD. Subclinical atherosclerosis, highlighted by the CAC score, is an indicator of increased CV risk, both in the general population and in individuals with CKD or diabetes. A CAC score > 100 justifies the use of high-intensity statins, and in certain situations, additional therapies such as aspirin, PCSK9 inhibitors, or treatments to reduce triglycerides [9,132]. In comparison to the 2023 ESC guidelines [19], which state that there is insufficient evidence to suggest that evaluating the CAC scores or intima–media thickness helps reclassify CV risk in individuals with T2D, the AHA recommends statins for all diabetic patients aged 40–75, regardless of the CAC values [9].

The recommendations for patients with stage B HF are based on lifestyle modification and initiation of pharmacological therapy to prevent or delay progression to HF stage C or D. The ACC/AHA guidelines provide recommendations for optimal HF management in stage B [60]. DM, obesity, and hypertension are comorbidities associated with asymptomatic left ventricular dysfunction and its progression to symptomatic HF. These conditions should be managed according to the current clinical practice guidelines [14,19,64] to reduce disease progression to symptomatic HF and decrease mortality. 

*Stage 4*: For patients with HF, irrespective of the LVEF, treatment with SGLT2is (empagliflozin, dapagliflozin, or sotagliflozin) is recommended due to their demonstrated efficacy (Table 1). Sotagliflozin is a dual inhibitor of sodium–glucose co-transporters 1 and 2 that, in the SOLOIST-WHF trial, reduced events among individuals with T2D hospitalized for HF who were treated during or soon after hospitalization. In this trial, the average LVEF was 35%, with most participants having HFrEF. Treatment with sotagliflozin was associated with a 29% reduction in worsening HF or CV death during an average 18 months of follow-up [135]. Moreover, in patients with T2D and CKD, with or without albuminuria, sotagliflozin resulted in a lower risk of the composite of deaths from cardiovascular causes, hospitalizations for HF, and urgent visits for HF than placebo [136]. Considering these results and the aggregate findings from SGLT inhibitor trials, the U.S. Food and Drug Administration recently granted an indication for sotagliflozin to reduce CV events in patients with HF [137].

Those with HFrEF require quadruple therapy, which includes RAASis (ACEi/ARNI), beta-blockers, steroidal mineralocorticoid receptor antagonists (MRAs), and SGLT2is, with the potential addition of a diuretic for congestion management. For patients with HFmrEF, along with SGLT2is, the integration of other components of quadruple therapy is recommended, if necessary [24]. Several clinical studies have started a new era in which SGLT2is join the foundational pillars of heart failure management across the EF spectrum [113,114,115,116,138]. These studies (Table 1) have demonstrated the impact of SGLT2is on ASCVD, reducing HF hospitalizations and CV mortality, independent of the diabetes status.

Moreover, finerenone demonstrated significant efficacy in reducing cardiovascular events and the worsening of HF in patients with HFpEF or HPmrEF [121], increasing its potential as one of the main pillars of therapy in patients with HFmrEF or HFpEF, along with SGLT2is.

Recent seminal trials have demonstrated the efficacy of incretin-based AOMs therapies in HFpEF patients, particularly in improving the quality of life in a clinically meaningful way. Moreover, combined data from the STEP heart failure trials (i.e., STEP-HFpEF and STEP-HFpEF DM) and the FLOW (patients with T2D and CKD) and SELECT (patients post ASCVD or peripheral arterial disease) trials showed that relative to placebo, semaglutide reduced the risk of the combined endpoint of cardiovascular death or HF by 31% [6,104,105]. Furthermore, tirzepatide in patients with HFpEF (Table 1) showed significant improvement in a composite of death from cardiovascular causes or worsening HF and change in health status in patients with HFpEF and obesity, which makes tirzepatide a suitable alternative to semaglutide for patients with HFpEF and obesity [6].

However, the potential benefits of GLP-1RAs and dual GIP/GLP-1 RAs in patients with HFrEF or ASCVD without excess weight/obesity remain unexplored, and further studies are needed to assess their effectiveness. SGLT2 inhibitors, GLP-1 receptor agonists, and dual GIP/GLP-1RAs play a significant role in managing CKM syndrome, offering broad benefits. These therapies help achieve optimal glycemic control, reduce body mass, lower BP, and reduce the risk of MACEs. They also support the cardiac and renal function, positively impacting HF, ASCVD, and CKD.

Although there are guidelines for each individual disease, they do not sufficiently address the interactions between them. Each of these professional society recommendations focuses on isolated organ systems, such as ASCVD, HF, and CKD, without addressing a unified management strategy. A transition from fragmented care to an integrated model adapted to the complexity of CKM syndrome, is proposed. Currently, an organ-centered approach leads to diagnostic overlaps, contradictory treatments, and polypharmacy, affecting patient adherence and clinical outcomes. Clear recommendations, unified guidelines, and training programs need to address all these conditions simultaneously, improving patient outcomes and quality of life.

## 9. Conclusions

Despite progress, care for patients with overlapping CKM diseases remains highly fragmented, often framed within individual specialties such as cardiology, nephrology, hepatology, and endocrinology, each focusing on a narrow aspect of disease progression. This fragmented approach leads to patient confusion and poor adherence to treatment plans, resulting in disease progression and poor outcomes. The definition of CKM syndrome in 2023 was a crucial step in addressing the integrated management of cardiovascular, renal, and metabolic comorbidities. This unified definition facilitates the recognition of CKM syndrome as a distinct clinical entity and encourages multidisciplinary collaboration across these domains. Clarifying the concept allows for a better understanding of the common and interconnected pathophysiological mechanisms between metabolic pathways, the heart, and the kidneys. Internal medicine professionals could provide holistic and patient-centered care across the entire CKM spectrum.

## Figures and Tables

**Table 1:** Recent studies involving drug options for patients with CKM syndrome.

Class	Name	Author, Year	Population, Follow-Up	Agent	Main Results with Studied Drug
**GLP1-RA**	STEP 1	Wilding, J.P.H. et al., 2021 [102]	1961 adults, no T2D, with BMI ≥ 30 kg/m^2^ or BMI ≥ 27 kg/m^2^ plus at least one weight-related comorbidity, 68 w	Once weekly s.c. semaglutide 2.4 mg	A mean weight loss of 14.9%
	STEP 2	Davies, M. et al., 2021 [103]	1210 adults with T2D, overweight, or obesity, 68 w.	Once weekly semaglutide, 2.4 mg, semaglutide 1.0 mg plus lifestyle interventions	Weight loss was 9.6% with semaglutide at a 2.4 mg dose and 7.0% with semaglutide at a 1.0 mg dose Additionally, greater reductions in HbA1c, baseline BMI, or glycemic control status
	STEP-HFpEF	Kosiborod, M.N. et al., 2023 [104]	529 adults with HFpEF (LVEF ≥ 50%) and obesity (BMI ≥ 30 kg/m^2^), on standard background therapies, 52 w.	Once weekly s.c. semaglutide 2.4 mg	The mean percentage reduction in body weight was −13.3% Secondary outcomes demonstrated positive changes in the 6 min walk distance test
	STEP-HFpEF DM	Kosiborod, M.N et al., 2024 [105]	616 patients with HFpEF and T2D, 52 w	Once weekly s.c. semaglutide 2.4 mg	The mean percentage reduction in body weight was −9.8% The change in the 6 min walk distance test was 14.3 m for semaglutide and 1.8 m for placebo
	FLOW	Perkovic, V. et al., 2024 [106]	3533 patients with T2D and CKD, defined by a sustained eGFR decline and albuminuria on standard care, 3.5 y	Once weekly s.c. semaglutide 1 mg	24% risk reduction of primary events (severe renal decline or CV events), 29% risk reduction of CV mortality, 18% risk reduction of MACEs, and risk reduction of all-cause mortality by 20%; reduction of the risk of new-onset or worsening HF by 27% Slowed the decline in kidney function, with a less steep annual rate of 1.16 mL/min/1.73 m^2^
	SOUL	McGuire, D.K. et al., 2025 [107]	9650 participants aged ≥50 years, with T2D and established CAD, cerebrovascular disease, symptomatic PAD, or CKD, 49.5 mo	Oral semaglutide, 14 mg o.d.	14% relative reduction in MACEs (CV death, non-fatal MI, and non-fatal stroke), primarily driven by a reduction in non-fatal MI
	STRIDE	Bonaca M.P. et al., 2025 [108]	792 patients with T2D and early symptomatic PAD, 52 w	Once weekly s.c. semaglutide 1.0 mg	A significant improvement in maximum walking distance (21%) vs. placebo (8%), pain-free walking distance, quality of life, ankle-brachial index, and disease progression
**dual** **GIP/GLP-1RA**	SURMOUNT-1	Jastreboff, A.M. et al., 2022 [109]	2539 adults, no T2D, with BMI ≥ 30 kg/m^2^ or BMI ≥ 27 kg/m^2^ plus weight-related complications, 72 w.	Once weekly s.c. tirzepatide (5, 10, or 15 mg) plus lifestyle changes	20.9% mean weight reduction with tirzepatide vs. 3.1% with placebo Improvements in all prespecified cardiometabolic measures were observed with tirzepatide
	SURMOUNT-2	Jastreboff, A.M. et al., 2024 [110]	938 adults with T2D and overweight or obese, 72 w.	Once weekly s.c. tirzepatide (5, 10, or 15 mg) plus lifestyle changes	The least-squares mean reduction in body weight at week 72 was −14.7% with tirzepatide, 15 mg, compared to −3.2% with placebo
	SUMMIT	Packer, M. et al., 2024 [111]	731 patients with HFpEF and obesity, with or without T2D, 60% with CKD, 104 w.	Once weekly s.c. tirzepatide (up to 15 mg)	38% risk reduction of death from CV causes or a worsening HF event. A significant improvement in KCCQ-CCS at 52 weeks was observed in the tirzepatide group
	SUMMIT Substudy	Packer, M. et al., 2025 [112]	731 participants with HFpEF and BMI ≥ 30 m^2^/kg, (60% with CKD), 52 w	Once weekly s.c. tirzepatide (up to 15 mg)	Tirzepatide improved renal function in patients with HFpEF, obesity, and CKD Tirzepatide increased eGFR at 52 weeks, assessed by both creatinine-based and cystatin C–based formulae
**SGLT2i**	DAPA-HF	McMurray, J.J.V. et al., 2019 [113]	4744 patients with symptomatic HFrEF and NYHA class II–IV (40% T2D), 18.2 mo	Dapagliflozin 10 mg o.d., in addition to the usual therapy	26% risk reduction of the primary composite outcome of worsening HF (hospitalization or an urgent visit resulting in intravenous therapy for HF) or death from CV causes HF hospitalizations were reduced by 30%, while CV death was reduced by 18%
	EMPEROR-Reduced	Packer, M. et al., 2020 [114]	3730 patients with HFrEF, NYHA class II–IV, median LVEF 27% (50% T2D), 16 mo	Empagliflozin 10 mg o.d., in addition to the usual therapy	25% risk reduction of the primary composite outcome of death from CV causes or hospitalization for HF The risk for the first HF hospitalization was reduced by 31%, and CV death by 8%
	DELIVER	Solomon, S.D. et al., 2021 [115]	6263 patients with HF and an LVEF of >40% (45% T2D), 2.3 y	Dapagliflozin 10 mg o.d., in addition to the usual therapy	18% risk reduction of the composite of worsening HF or CV death. Worsening HF was reduced by 21%, and CV death by 12%. Total events and symptom burden were lower in the dapagliflozin group than in the placebo group
	EMPEROR-Preserved	Anker, S.D. et al., 2021 [116]	5988 patients with HF, LVEF > 40% (49% T2D), 26.2 mo	Empagliflozin 10 mg o.d., in addition to the usual therapy	21% risk reduction of the composite of CV death or hospitalization for HF 27% risk reduction of HF hospitalizations
	DAPA-CKD	Heerspink, H.J.L. et al., 2020 [117]	4304 pts (67.5% T2D) with CKD (eGFR 25–75 mL/min/1.73 m^2^, UACR 200–5000 mg/g, 2.4 y	Dapagliflozin 10 mg o.d.	39% risk reduction for the primary endpoint (eGFR decline ≥ 50%, ESKD or renal/cardiovascular death); 5.3% absolute reduction in primary event rate; 29% reduction in HF hospitalizations; 31% reduction in all-cause mortality Efficacy across all subgroups, including diabetic (36% risk reduction) and non-diabetic (50% risk reduction) patients
	EMPA-KIDNEY	Herrington, W. et al., 2023 [118]	6609 pts (46.2% T2D) with CKD (defined by an eGFR of 20–45 mL/min/1.73 m^2^ or 45–90 mL/min/1.73 m^2^, UACR 200–5000 mg/g, 2y.	Empagliflozin 10 mg o.d.	28% reduction in CKD progression or renal/cardiovascular death; 3.8% absolute reduction in primary event rate; 36% risk reduction in patients with T2D; 18% risk reduction in patients without T2D
** ns-MRA **	FIDELIO-DKD	Agarwal, R. et al., 2022, [119]	5734 patients with CKD and T2D, 2.6 y	Finerenone 20 mg (eGFR ≥ 60 mL/min/1.73 m^2^); 10 mg (eGFR 25–60 mL/min/1.73 m^2^)	Decreased CKD progression by 18% and CV mortality and morbidity by 14%
	FIGARO-DKD	Filippatos, G. et al., 2022, [120]	7437 patients with CKD and T2D, 3.4 y	Finerenone 20 mg (eGFR ≥ 60 mL/min/1.73 m^2^); 10 mg (eGFR 25–60 mL/min/1.73 m^2^)	Reduced CV mortality and morbidity by 13% and CKD progression by 13%
	FINEARTS-HF	Solomon, SD. et al., 2024, [121]	6001 patients with symptomatic HF and LVEF ≥ 40%, 32 mo	Finerenone 40 mg daily (20 mg if eGFR < 60 mL/min/1.73 m^2^).	Reduced HF events by 18% and cardiovascular death by 7%. Kidney disease progression was reduced by 23%

T2D, type 2 diabetes mellitus; BMI, body mass index; w, weeks; s.c., subcutaneously; HFpEF, heart failure with preserved ejection fraction; LVEF, left ventricle ejection fraction; CKD, chronic kidney disease; eGFR, estimated glomerular filtration rate; y, years; HF, heart failure; CAD, coronary artery disease; PAD, peripheral artery disease; HFrEF, heart failure with reduced ejection fraction; NYHA, New York Heart Association; mo, months; CV, cardiovascular; UACR, urinary albumin to creatinine ratio.

## Data Availability

Not applicable.

## Supplementary Materials

The following supporting information can be downloaded at: https://www.mdpi.com/article/10.3390/jcm14082833/s1, Figure S1: The spectrum of Cardiovascular–Renal–Metabolic diseases; Table S1: Diagnostic criteria for prediabetes and diabetes according with WHO and ADA in non-pregnant subjects; Figure S2: Residual cardiovascular risk calculators; Figure S3: The management in CKM syndrome stage 0. Figure S4: Management strategies in CKM stage 1.

## Abbreviations

The following abbreviations are used in this manuscript:

AHA	American Heart Association
ACC	American College of Cardiology
CKM	Cardiovascular–Kidney–Metabolic
CVDs	Cardiovascular Diseases
CKD	Chronic Kidney Disease
ESKD	End-Stage Kidney Disease
MetS	Metabolic Syndrome
HF	Heart Failure
LVEF	Left Ventricular Ejection Fraction
HFpEF	Heart Failure with Preserved Ejection Fraction
HFmrEF	Heart Failure with Mildly Reduced Ejection Fraction
HFrEF	Heart Failure with Reduced Ejection Fraction
WHO	World Health Organization
DM	Diabetes Mellitus
T1D	Type 1 Diabetes
T2D	Type 2 Diabetes
CAD	Coronary Artery Disease
DKD	Diabetic Kidney Disease
eGFR	Glomerular Filtration Rate
BMI	Body Mass Index
SDOH	Social Determinants of Health
FFAs	Free Fatty Acids
HDL	High-Density Lipoprotein
SNS	Sympathetic Nervous System
AGEs	Advanced Glycation End Products
KDIGO	Kidney Disease Improving Global Outcomes
ASCVD	Atherosclerotic Cardiovascular Disease
ADA	American Diabetes Association
UACR	Urinary Albumin-to-Creatinine Ratio
ESC	European Society of Cardiology
Lp(a)	Lipoprotein(a)
CAC	Coronary Artery Calcium
GLP-1RAs	Glucagon-Like Peptide 1 Receptor Agonists
GIP/GLP-1RA	Glucose-Dependent Insulinotropic Polypeptide/Glucagon-Like Peptide-1 Receptor Agonist
SGLT2is	Sodium-Glucose Cotransporter-2 Inhibitors
MACEs	Major Cardiovascular Events
ACEi	Angiotensin-Converting Enzyme Inhibitor
ARB	Angiotensin II Receptor Blocker
MRAs	Mineralocorticoid Receptor Antagonists
SCORE2	Systematic Coronary Risk Evaluation 2
RASS	Renin–Angiotensin–Aldosterone System
RAASi	Renin–Angiotensin–Aldosterone System Inhibitor
