# Supplementary material for: Cardiovascular–Kidney–Metabolic Syndrome: A New Paradigm in Clinical Medicine or Going Back to Basics?
Source: J Clin Med. 2025 Apr 19;14(8):2833. doi: 10.3390/jcm14082833 (PMC12028252; doi:10.3390/jcm14082833)
Supplement: Supplementary file 1 [file jcm-14-02833-s001.zip › jcm-3552801-supplementary.pdf]

Supplementary material

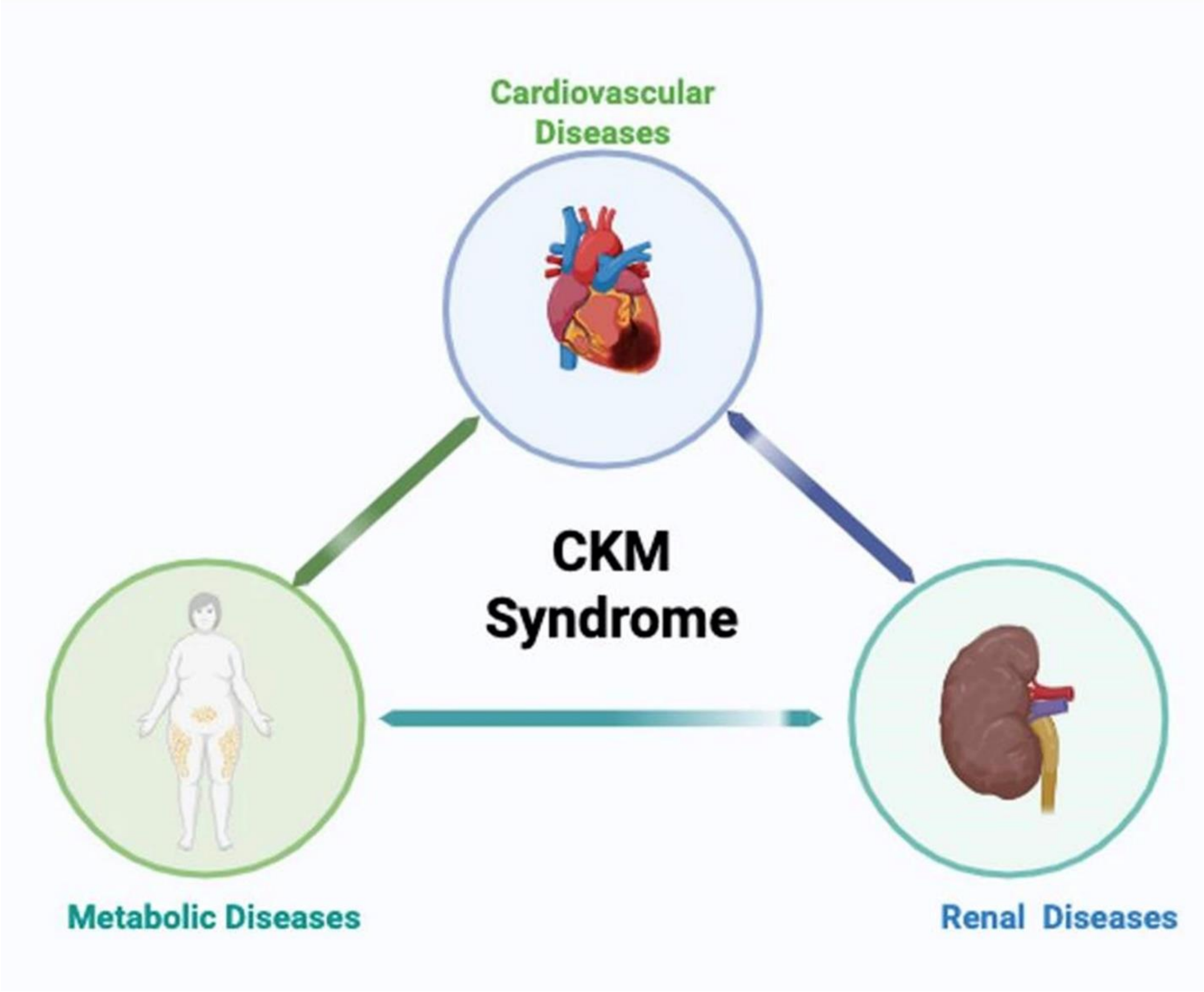

Supplementary Figure S1. The spectrum of Cardiovascular–Renal–Metabolic diseases.

Supplementary Table S1. Diagnostic criteria for prediabetes and diabetes according to WHO and ADA in non-pregnant subjects.

Criterion*	Prediabetes	Diabetes
A1C**	5.7-6.4% (39–47 mmol/mol)	≥6.5% (≥48 mmol/mol)
FPG	100 mg/dL (5.6 mmol/L) to 125 mg/dL (6.9 mmol/L) representing IFG	≥126 mg/dL (≥7.0 mmol/L)
OGGT	140 mg/dL (7.8 mmol/L) to 199 mg/dL (11.0 mmol/L) representing IGT	2-h PG ≥200 mg/dL (≥11.1 mmol/L)

*In the absence of unequivocal hyperglycemia, diagnosis requires two abnormal results from different tests which may be obtained at the same time (e.g., A1C and FPG), or the same test at two different time points; **The test should be performed in a laboratory using a method that is National Glycohemoglobin Standardization Program certified and standardized to the Diabetes Control and Complications Trial assay; FPG, fasting plasma glucose (fasting defined as no caloric intake for at least 8 hours); IFG, impaired fasting glucose; OGGT, oral glucose tolerance test, performed as described by the WHO, using a glucose load containing the equivalent of 75 g anhydrous glucose dissolved in water; IGT, impaired glucose tolerance.

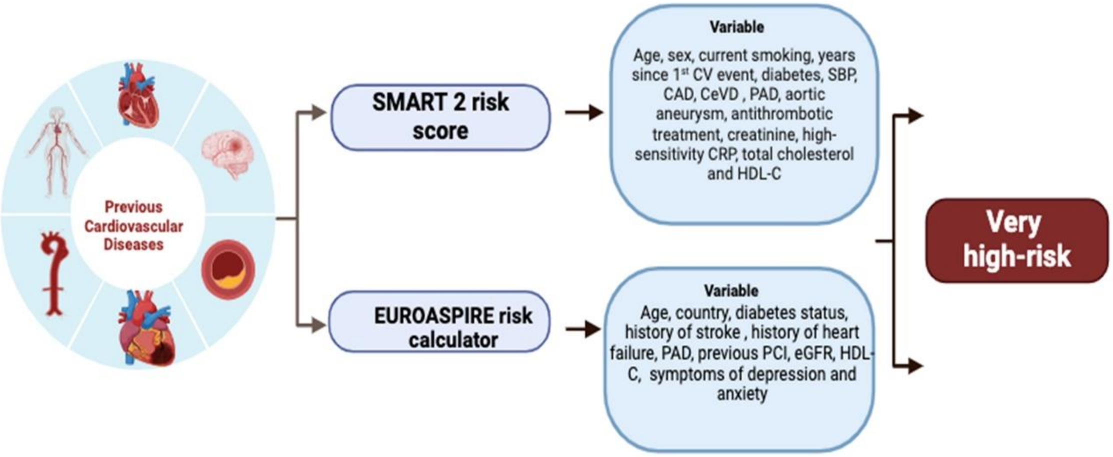

Supplementary Figure S2. Residual cardiovascular disease risk calculators.

SBP, systolic blood pressure; CAD, coronary artery disease; CeVD, cerebrovascular disease; CRP, C-reactive protein; CV, cardiovascular; eGFR, glomerular filtration rate; HDL-C, HDL cholesterol; PAD, peripheral artery disease; PCI, percutaneous coronary intervention

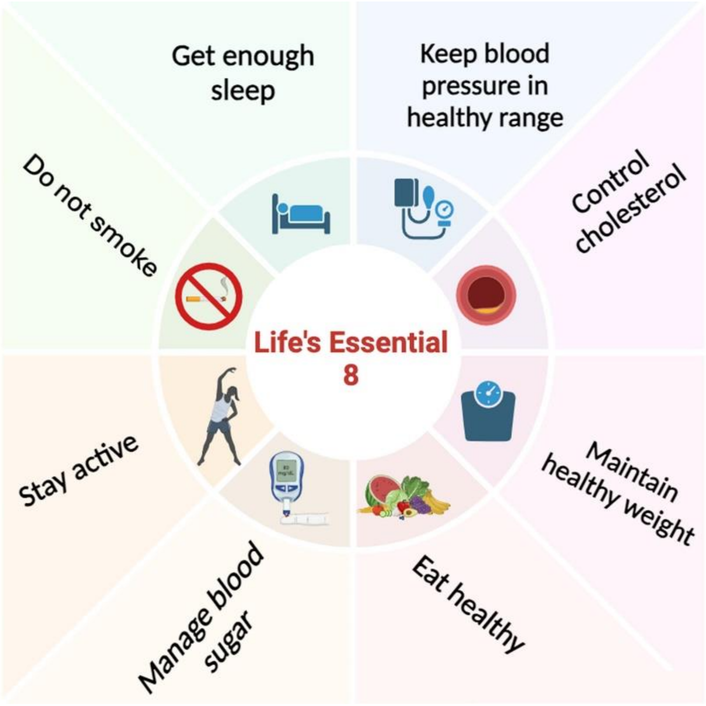

Supplementary Figure S3. The management in CKM syndrome stage 0. Life's Essential 8 includes the 8 components of cardiovascular health: healthy diet, participation in physical activity, avoidance of nicotine, healthy sleep, healthy weight, and healthy levels of blood lipids, blood glucose, and blood pressure.

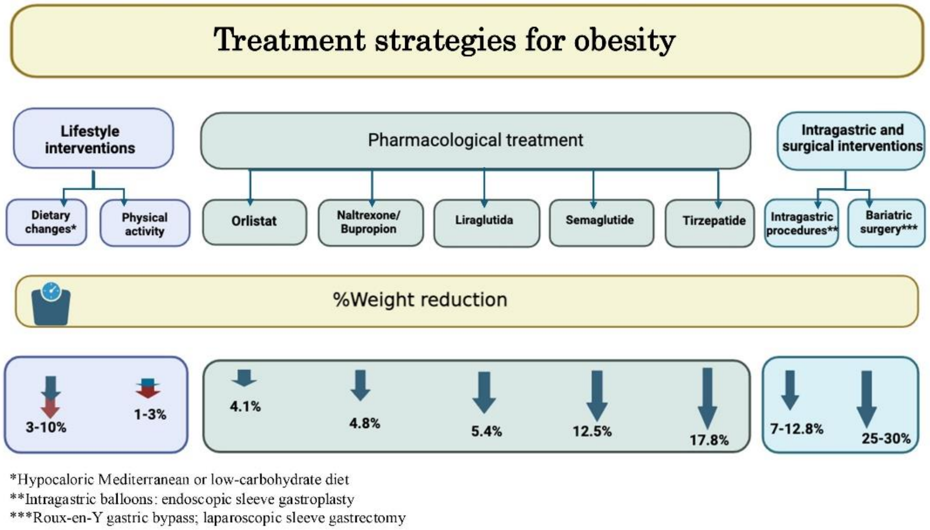

Supplementary Figure S4. Management strategies in CKM syndrome stage 1 (with expected effects of weight loss interventions on body weight, adapted from [14]).
